# Supplementary material for: Are carbon emissions trading and green financial instruments synergistic? -Comprehensive quantitative research based on content analysis
Source: PLoS One. 2024 Mar 7;19(3):e0298601. doi: 10.1371/journal.pone.0298601 (PMC10919601; doi:10.1371/journal.pone.0298601)
Supplement: S1 Data — (ZIP) [file pone.0298601.s001.zip › Data/地级市Z_F工作报告.zip!/╔╜╬≈/╨├╓▌╩╨/└┤╫╘╣1⁄2╓┌║┼í╛┬φ┐╦╩2╛▌═°í┐_2014.docx]

各位代表：

　　现在，我代表市人民政府向大会报告工作，请予审议，并请市政协委员和其他列席会议人员提出意见。

　　一、2013年工作回顾

　　一年来，市政府在省委、省政府和市委的正确领导下，坚持主题主线，坚持综改统领，坚持稳中求进，认真实施“3581”发展战略，扎实推进各项工作，总体完成了年初确定的目标任务。

　　（一）积极应对下行压力，经济保持平稳发展。

　　坚决执行中央宏观调控政策和省委、省政府、市委决策部署，及时制定、认真实施促进经济平稳、较快增长9条意见，推动煤炭经济可持续增长10条意见，支持中小微企业发展14条意见等文件。

　　加大项目攻坚力度。坚持“八位一体”推进项目建设，储备、签约、落地、开工、建设、投产任务提前1个月完成，省、市两级重点工程完成投资814亿元。加快大西客运专线忻州站建设。灵河高速浑源王庄堡至繁峙段建成通车。加快五台山机场建设，与省民航机场集团签署托管协议。云中水厂并网运行。重大基础设施项目完成投资249亿元，为长远发展积蓄了后劲。

　　加快重点产业发展。加快煤炭企业达产达效，完成产量6161.5万吨、发运量5318万吨。神达集团计划单列获批运行。神华河电一期等电力项目加快建设，华润宁武低热值煤发电项目取得“路条”。加快打造千万千瓦级风电基地。同德氧化铝一期项目进展加快。蓝天公司着力打造节能环保锅炉制造基地，久力公司首开全市矿山废弃物转化利用先河，节能环保产业成为发展的新亮点。五台山加快改造提升，芦芽山、雁门关加快创建5A级景区。服务业发展提速，占地区生产总值的比重超过40%。非煤产业占规模以上工业增加值比重超过60%。

　　切实服务企业生产经营。暂停提取有关费用，减轻煤炭企业负担。免征11436户企业的所得税和增值税、625户小微企业的营业税。“营改增”试点惠及企业1000余户。新增中小企业担保资金3300万元。市财政投入3000万元，吸收社会资本7000万元，设立中小企业创业投资基金，支持中小微企业发展。全市财政注入“助保贷”项目风险补偿金4531万元，带动企业缴纳助保金1698万元，累计贷款37960万元，惠及企业83户。推动民营企业转型升级，全年新增中小微企业1212个、营业收入超亿元的“小巨人”企业16个。中小微企业成为促进就业、推动经济发展的重要力量。

　　一年来，在困难的情况下，全市经济社会发展稳中有进，多项经济指标增幅全省靠前。全年地区生产总值完成654.7亿元，增长9%；固定资产投资完成815.2亿元，增长24.8%；社会消费品零售总额完成246亿元，增长14.2%；公共财政预算收入完成73.7亿元，增长13.1%；城镇居民人均可支配收入20324元，增长9.9%；农民人均纯收入5426元，增长13.6%。居民消费价格涨幅3%，城镇登记失业率3.17%。9项约束性指标全部完成。

　　（二）精心实施第二个“大干城建年”，市域城镇化取得预期成效。

　　努力改善城镇面貌。忻州城区总投资22.9亿元的29条城市道路全部竣工通车，忻顿路桥建成通车，云中河景区工程和9条主干道绿化基本完成，“五馆一院”加快建设，中心城市承载能力和服务功能大幅提升。108国道城区过境改线竣工通车，形成城市东环线。各县（市）扩容提质步伐加快，列入全省“百镇建设”的示范镇建设任务顺利完成。全市市政公用设施建设投资完成年度计划的近1.5倍，城镇化率提高1.5个百分点以上。

　　认真规范建设市场秩序。顺应群众呼声，维护建设成果，开展中心城区“三项集中整治”。创新城市垃圾处理、商铺门店卫生管理，为城区600余名环卫工人提供免费早餐。健全查处违法建设长效机制，形成市区一体、露头就打、源头防范的新格局，城市建设步入良性轨道。启动城中村改造试点。启用忻州市数字化城市管理系统，主要区域实现了管理数字化、内容网络化、空间可视化。

　　稳步提高城镇化质量。全市16座污水处理厂全部达标运行，新建6座垃圾处理场全部完工；忻州城区新增供热面积150万平方米，集中供热普及率提高1.16个百分点；理顺城区公交运营体制，服务质量有所提升；启动城区和保德县创建省级园林城市工作，城区绿化覆盖率提高6.5个百分点；在城区布局近300万平方米的房地产开发和商贸项目，促进服务业加快发展，努力创造宜居宜业的环境。

　　（三）认真做好“三农”工作，农民收入持续较快增长。

　　不断巩固农业基础。认真落实各项强农惠农富农政策，着力调动农民生产积极性。1座小型病险水库和3座病险水闸除险加固任务完成，推进灌区节水改造，农田实灌面积超过190万亩。全市粮食总产33.9亿斤，再创历史新高。

　　加快推进农业产业化。新发展温室大棚1.1万亩，建设特色现代农业示范园区111个，新发展“一村一品”示范村273个，着力提升8个省级“一县一业”县建设水平。农产品加工企业销售收入突破50亿元，增长30%以上。新认证“三品”83个，新认证地理标志农产品5个。羊饲养量达到525万只，正在成为农民致富的重要支柱。创新农业生产经营体制，新增农民专业合作社1375个。

　　全力推进扶贫开发。狠抓“百企千村”产业扶贫开发工程，在全省率先举办项目推介签约会，省属9户帮扶企业全部进驻，成立农业开发公司11个，前期工作扎实推进。开工建设4万人的易地扶贫移民搬迁房，主体完工率82.3%。持续开展“千名干部进企业、万名干部下农村”活动，扎实做好机关定点扶贫工作。全市扶贫开发重点县农民人均纯收入突破5000元，全年减贫人口10万余人。

　　认真办好农村“五件实事”。改造农村困难家庭危房11720户，按计划推进特困群众易地搬迁，新建、改扩建村级幼儿园60所，为农村配备保洁员8729人、保洁车3758辆，为3591个村安装太阳能路灯71773盏，完成行政村街道亮化任务。开展两轮“五个全覆盖”工程回头看，努力巩固惠民成果。

　　（四）大力改善生态环境，美丽忻州建设迈出新步伐。

　　狠抓节能减排。试行能耗交易机制，全市万元GDP能耗首次实现按季达进度，能耗总量偏小制约发展的瓶颈开始破解，提前半年完成淘汰落后产能任务。深入开展创模工作。市级创模规划通过环保部评审，县（市）创建省级环保模范城市工作全面加快。积极开展大气污染防治，忻州城区二级以上天数达到287天，空气质量稳定达到国家二级标准。

　　狠抓集成创卫。出台创建国家卫生城市全覆盖工作方案，提出再用三年时间实现县城和重点镇创建国家卫生城镇全覆盖。五台县、宁武县、保德县以及繁峙县砂河镇、五台县台怀镇、定襄县河边镇创卫工作通过验收，其他各县创卫前期工作扎实推进。全市通过验收发文的全国卫生城镇达到11个，位居地级市全国第二位、全省第一位。

　　狠抓造林绿化。完善林业发展总体布局，巩固扩大林改成果。扎实推进“两山”造林、“两网”绿化、“两林”富民、“两区”增绿，完成营造林61.2万亩，为建设美丽忻州奠定了坚实基础。

　　（五）切实保障、改善民生，社会事业不断发展。

　　坚持把就业作为民生之本。认真落实高校毕业生就业各项政策，引导高校毕业生积极创业和到基层、中小企业就业，公开招录公务员199名，应届毕业生就业率得以提高。加强公益性岗位管理，在岗人员达到11625人。开展就业困难人员帮扶工作，加强创业培训，倡导创业就业。全年城镇新增就业3.92万人，转移农村劳动力4.3万人。

　　全面加强社会保障。城乡居民基础养老金每人每月增加10元，企业退休人员基本养老金提高10%。城镇居民医保和新农合财政补助标准提高40元。出台城乡居民低保管理办法和低收入群众认定办法，城乡低保标准每人每月分别提高30元、24元。加大社会救助力度。认真落实退役士兵安置18条规定，着力完成安置任务。

　　切实提高城乡居民收入。第三次提高全市公务员津贴补贴标准，全部兑现事业单位人员绩效工资。认真执行全省最低工资标准，切实提高低收入职工收入。提高机关、企事业单位及离退休人员取暖补贴标准，提前完成96万吨农户取暖煤发放任务。全面推行农民工工资支付登记卡管理办法，着力破解农民工工资拖欠问题。实施“一元菜”工程，居民菜价得到有效平抑。

　　努力改善城乡居民居住条件。新开工各类保障性住房17754套，基本建成12106套；城区春节前一次公开摇号分配8030套。3000户农村住房抗震改建工程基本竣工。加强房地产市场监管，落实开发项目配套设施建设规定，房地产开发水平不断提高。

　　统筹城乡社会事业发展。扎实推进义务教育学校标准化建设，实施普通高中“四化一改”工程，扩大中等职业教育覆盖面，新建、改扩建公办幼儿园36所，努力推动教育公平。县级公立医院综合改革试点稳步推进，忻州市人民医院主体完工，市儿童医院完成选址。实施“十大文化工程”，县级“三馆一院”加快推进，戏曲电影《黄河管子声》、数字电影《徐向前》开机，遗山墓园重修开园，群众文化活动多姿多彩。全民健身活动蓬勃开展，忻州挠羊赛成为山西唯一入选首届全国体育非物质文化推广与保护遗产项目，我市运动员梁磊蝉联全运会自由式摔跤120公斤级项目冠军。

　　加强基层治理体系建设。扎实推进平安忻州建设，社会治安持续好转。市公安局获得第八届全国“人民满意的公务员集体”荣誉称号。认真做好食品药品安全工作。全面贯彻民族宗教政策。切实加强预备役建设和国防后备力量建设。

　　人口计生、外事、侨务、统计、应急、人防、气象、地震、文物、史志、档案等工作得到新加强，老龄、残疾人、红十字会等事业取得新进展。

　　（六）坚持不懈狠抓安全生产，本质安全水平得到提升。

　　牢记“三个决不过高估计”、“三个敬畏”，始终以如临深渊、如履薄冰的心态做好安全生产工作。坚决落实政府监管责任，努力推行综合监管、部门监管、专门监管、行业监管、属地监管责任的全覆盖、全天候和立体化。以落实政府监管责任推动企业落实主体责任，健全企业安全生产机构、安全风险预控机制、应急救援体系，切实夯实安全生产基础。紧紧抓住煤矿安全，高度重视非煤矿山安全，强化尾矿库安全监管，扎实开展打非治违，深入开展专项检查，认真做好防洪防汛、道路交通、地质灾害防治等工作，确保重点行业、重点领域安全。深入开展安全生产大检查和“安全知识进家庭、进学校、进社区”活动，强化安全生产培训和警示教育成效，努力提高全社会安全意识。全市安全生产死亡人数控制在省定指标的65.2%，煤矿百万吨死亡率0.026。

　　（七）着力推进改革开放，发展的动力活力不断增强。

　　加快推进转型综改区建设。出台三年实施方案和2013年行动计划以及重大产业转型项目认定办法和若干优惠政策，实质性推进转型综改区建设。加快建设“三个门户”、打造“三个集散地”、发展“六大经济板块”。初步建立“五规合一”统筹协调机制。城乡建设用地增减挂钩等改革深入推进。晋商银行、交通银行进驻，忻府区农商行成立，五台农商行忻州秀容小微专营支行开业，忻州中小微企业融资服务中心、忻府区秀都村镇银行等筹建，金融创新取得新的进展。完成矿产资源税费制度改革等5个课题研究。

　　积极推进企业改革。钨丝厂改革和忻通公司、水务集团重组基本完成，云河集团、云马公司、云瑞公司转型和破产工作顺利推进。钨丝厂、云马公司、云瑞公司三户企业职工安置工作基本完成。民营企业致力打造经营、管理和技术三个团队，加快现代企业制度建设。蓝天公司技术中心成功创建国家级企业技术中心，12户企业技术中心通过省级认定，1户企业被认定为省级行业技术中心，新认定市级企业技术中心66户，实现了我市国家级企业技术中心的零突破和市级企业技术中心全覆盖。

　　深化行政审批制度改革。全面完成清理、对接省级下放和取消行政审批事项等工作。市级行政审批事项取消5项、下放6项、调整2项、压减至107项，审批时限比法定时限减少三分之二。

　　努力扩大对外开放。制定出台招商引资奖励办法，坚持以企引企、以商招商，开展园区招商和产业链招商，不断拓展招商渠道和引资载体。积极参加中博会、农博会、文博会等重大展会，不断深化与发达地区的经贸文化合作，全年签约项目188个、总投资1642.7亿元，招商引资到位资金突破500亿元。

　　（八）坚持从严治政，政府自身建设进一步加强。

　　严格执行“八项规定”，坚决反对“四风”，扎实做好群众路线教育实践活动准备工作。认真执行市人大及其常委会的各项决议，主动接受市人大的法律监督和市政协的民主监督。自觉向人大报告工作、向政协通报工作，积极支持市政协参政议政。全年共办理人大代表建议87件、政协委员提案349件。继续开展“给人民汇报、向人民问计、请人民评议”活动，自觉把政府工作置于全社会监督之下。深入开展“六五”普法，严格规范性文件合法性审查，依法有效化解行政争议，强力推进法治政府建设。强化行政执法监督，坚决纠正违法行为。严格控制一般性支出，努力压缩“三公”经费支出，节俭办文、办会、办事，完成公车和办公用房清理规范工作。不断强化行政监察和审计监督。制定《忻州市人民政府工作规则》，推动政府工作制度化、规范化。

　　各位代表，回首去年工作，成绩来之不易。这是市委总揽全局、坚强领导的结果，是市人大、市政协大力支持、有效监督的结果，是全市人民群策群力、艰苦奋斗的结果。在此，我代表市人民政府，向全市人民、驻忻部队指战员、武警官兵、公安民警和各驻忻单位，向各民主党派和人民团体，向所有关心、支持忻州发展进步的朋友们，致以崇高的敬意，表示衷心的感谢！

　　我们清醒地知道，我市经济社会发展还存在不少困难和问题，主要是：工业经济运行困难，经济下行压力加大，财政增收难度加大；经济总量仍然较小，带动性强的大项目、好项目不多；传统产业远未做大做强，新型产业发育严重不足；民生和社会事业欠账仍然较多，农民增收特别是扶贫开发困难很大；创新创业的氛围不够浓厚，发展的环境不够优化；综改试验区建设成效不够明显，深化改革、创新攻坚没有取得重大突破；政府工作还存在不少缺点，距人民群众的期望还有很大差距。这些问题，有的是发展过程中产生的，有的是工作不到位造成的。对存在的问题，政府要先从自身找原因，直面矛盾，勇于担当，勤勉工作，毫不懈怠，决不辜负组织和人民的厚望。

　　二、2014年工作安排

　　2014年是全面贯彻落实党的十八大和十八届三中全会精神、扎实推进转型跨越发展的关键一年。做好今年的政府工作，要坚持以中国特色社会主义理论体系为指导，准确把握稳中求进和改革创新的总要求，创新实施“3581”发展战略，以转型综改试验区建设为统领，全面深化改革，抓好重点突破，扎实推进经济、政治、文化、社会、生态文明建设，确保实现科学发展、转型跨越、赶队前行、进位争先的年度目标，加快建设富裕文明、开放和谐、充满活力的新型工业旅游城市，为忻州与全省同步全面建成小康社会而努力奋斗。

　　今年经济社会发展的主要预期指标是：地区生产总值增长9%，固定资产投资增长20%，社会消费品零售总额增长14%，公共财政预算收入增长11%，城镇居民人均可支配收入增长12%左右，农民人均纯收入增长13%以上，城镇新增就业3.7万人，城镇登记失业率控制在4.2%以内，居民消费价格总水平涨幅控制在3.5%左右。要加强对各项主要指标的统筹平衡，完成省下达的各项约束性指标。

　　这里，着重对经济增长加以说明。不唯GDP，不是不要GDP，而是对GDP的质量和效益提出了更高的要求，要求经济增长必须处于合理区间。忻州作为后发地区，只有保持一定的增长速度，才能实现赶队前行。我们只要奋发努力，这个增长速度就一定能够实现；也只有实现这个目标，才能稳定就业、保障支出。

　　做好今年政府工作，要以深化改革为强大动力，以项目建设为主攻方向，以改善民生为根本目的，统筹兼顾，突出重点，务求实效。

　　（一）坚持以综改区建设为统领，加快赶队前行、进位争先。

　　大力开展“转型综改攻坚年”活动。认真落实转型综改三年实施方案和2014年行动计划，在抓好“四个重大”上下功夫，推进20项重大改革、30个重大事项、30个重大项目、3项重大课题。认真做好“三县、两园、一县一企”等综改试点工作，切实发挥引领示范作用。

　　努力加快以转化促转型的步伐。把综改试验区建设的着力点放在服务转化需要、统筹推进改革上，推动现有企业转化升级。坚持以扩张促转化，加快做大总量，发挥规模效应。抓紧提升煤炭生产、洗选能力，尽快达到批复产能；抓紧完成煤电园区规划，全力争取低热值煤发电项目，加快推进河保偏、原宁静煤电基地建设，确保“十二五”末全市电力装机达到1000万千瓦。坚持以延伸促转化，开发上下游产品，延长产业链条。重点发展煤—电、煤—化、煤—焦—化、铁矿—铁精粉—钢（铸）、铝矿—氧化铝—铝型材等产业链，努力构建全产业链。坚持以提升促转化，加快企业技术创新，增强市场竞争能力。坚持以衍生促转化，加快产品系列开发和新产业培育，实现一业为主、多元做大。切实提高矿产资源、旅游资源、农业资源的开发转化利用率，尽快把资源优势转化成优势产业；进一步做大煤炭物流业，加快定襄永旺物流园区建设，推动电子商务建设，尽快把区位优势转化成商贸物流产业；推动温泉休闲产业转型升级，大力发展适应消费需求的新型服务业，尽快把市场需求转化成现实生产力。推进产业集聚发展。加快建设“三个门户”，发挥桥头堡作用；加快打造“三个集散地”，推动旅游目的地建设；加快发展“六大经济板块”，促进县域经济融合发展；切实提升“一园六企”建设水平，推动新上产业项目向园区集中。

　　不断扩大对外开放。做好中博会、能博会等参展工作，提升招商引资水平。创新开发区和各类园区管理体制，积极发展“飞地经济”，加快产业集聚区建设。加快融入大太原都市圈、晋陕蒙金三角、环渤海经济圈，深化与长三角、珠三角、东三省等地区的合作。进一步引才引智，力争在引进国内国际500强企业上取得新突破。

　　深化金融创新。积极引导民间资本进入金融领域，推动农村信用社改制，大力发展村镇银行、融资性担保公司等金融机构。着力推动金融创新，切实提高全市存贷比。建立银企对接长效机制，定期组织银企现场对接，设立融资服务电子平台。鼓励支持优质企业通过票据、债券、股权、上市等方式实现直接融资。不断优化金融生态环境，严厉打击各类非法集资活动，规范小额贷款公司放贷行为，有效防范金融风险，为金融资本支持地方经济发展创造条件。

　　改革永无止境，转型重在行动。我们要牢固树立综改意识，以综改的视角看问题，以综改的办法解难题，一切不利于发展的体制机制弊端都要坚决改，一切有利于发展的措施都要大胆试，努力开创全市转型综改区建设的新局面。

　　（二）认真解决经济运行中的突出问题，促进经济健康协调可持续发展。

　　搞好经济运行监测。不断完善市县两级经济运行监测联席会议和工业经济运行监测联席会议制度，切实发挥预警和参谋作用，牢牢掌握经济工作主动权。

　　着力服务企业生产经营。认真落实清费立税改革，严格矿产资源规费集中征收管理，严禁以任何名义向任何企业拉赞助、搞摊派。保障企业资金链安全。发挥中小企业创业投资基金作用；推动中小企业“助保贷”项目全覆盖，力争贷款企业达到100户以上，贷款规模达到5亿元以上；提升企业财务管理水平，完善企业资产凭证，为融资创造更好条件。鼓励企业技术创新。支持蓝天公司国家级技术中心建设和同德化工申报国家级技术中心，扩大省、市级技术中心的覆盖面。

　　大力促进企业健康发展。精心实施中小微企业成长工程，加快资源型企业转型项目建设，新培育中小微型企业1000个、年营业收入超亿元的“小巨人”企业10个，新上转型发展项目100个。做好服务企业上市工作。

　　不断扩大消费。鼓励信息消费和新兴服务类消费，支持发展社区便民早餐、洗浴等生活服务业，完善家政服务网络体系，加快“15分钟便民商圈”建设。推进农超对接，加快农产品市场建设，降低流通成本。规范消费市场秩序，让人民群众安全消费、放心消费。

　　（三）不断引深项目建设攻坚战，夯实稳增长的基础。

　　狠抓重点工程建设。继续坚持“八位一体”抓项目，统筹基础设施、产业发展、城镇化和生态环保、民生事业等重点领域投资，保持固定资产投资较快增长。今年安排省、市两级重点工程471项，年内计划完成投资729亿元。发挥好政府投资的引导作用，完工大西客专忻州段，加快原神、繁五高速公路建设，继续推进国道108线神堂堡至砂河段一级公路改建、集中连片特困地区县乡公路改造，加快五台山机场建设，抓好山西大水网忻州项目区建设。

　　加快重大产业发展。抓住新一轮国家即将实施一批煤电、煤化工项目的机遇，努力布局几个大型、特大型煤电、煤化工项目。开工建设华润宁武、忻州广宇等两个电厂项目，加快神华国能二期2×66万千瓦、河曲电厂三期2×100万千瓦项目前期工作；全力服务潞安五寨煤制油、阳煤岢岚煤制气项目，促进及早开工建设；加速推进阳方口煤炭洁净化循环经济工业园区规划，力争煤制烯烃项目及早入园；加快新石焦化200万吨焦炭及化产品回收项目；加速推进北辛窑等重点煤矿建设，同步规划洗煤厂项目，建设采选一体化千万吨级矿井；加快准池、准朔沿线集运站建设，推动煤炭运销产业稳定发展；加快同德氧化铝项目建设，启动信发氢氧化铝项目；加快久力尾矿砂制肥料、制微晶石项目建设，完善山西泰达等水泥项目手续；加快蓝天公司高效节能煤粉锅炉升级换代，不断占领技术制高点；加快推进忻州开发区忻通刮板机、美新液压支架和采煤机项目，带动煤机装备和维修基地建设。通过扭住重大项目，坚持大项带动，切实增强工业经济后劲。

　　加快工业园区建设。支持忻州经济开发区扩区，不断优化发展环境，完善基础设施，激发内部活力，吸引外来投资，培育支柱产业，搞好示范引领。大力提升原平循环经济示范区等10大市级工业园区建设水平，推进繁峙砂河航空产业园建设，真正把园区打造成产业集聚发展的载体。

　　加快新型服务业发展。认真落实支持服务业发展各项政策，积极发展现代物流、研发设计、检验检测等生产性服务业，大力发展育幼养老、家政服务、健康休闲等生活性服务业。加快提升五台山、芦芽山、雁门关、老牛湾等景区水平，积极打造顿村—奇村—汤头—云中河景区温泉休闲度假养生旅游集散地，促进全市旅游产业转型升级。

　　着力提高项目建设质量。立足各县（市、区）发展基础和资源禀赋，对重大产业建设项目实行三级分类考核，发挥好考核导向作用，增强考核的科学性。加强新建项目经济、社会和环保评估，把好项目论证和投资合作者选择关，严格市场准入标准。继续开展项目观摩，不断引深项目建设攻坚战。

　　（四）继续做好“三农”工作，切实增加农民收入。

　　稳定发展粮食生产。认真贯彻中央1号文件，调动农民种粮积极性。加快牧马河等灌区节水改造和“一村一井”项目建设，完成14座中小型水库专项应急除险加固工程，做好小型水库重建工作，新解决4万农村人口饮水安全问题。确保全市耕地保有量和基本农田数量不减少、质量有提高，完成土地开发年度任务。推进中低产田改造和粮食高产工程，改造中低产田24万亩，建设万亩粮食高产示范区50个，不断提升农业综合生产能力，切实保障粮食安全。搞好粮食收储，重视解决“卖粮难”问题。

　　加快推进农业产业化。坚持以工业化理念、产业化路子推动农业升级改造。打造知名品牌。大力培育国家级、省级农产品知名品牌，新增认证“三品”50个以上；加快推进以豆面、莜面、荞面为主的杂粮主食化，以亚麻油、糯玉米、马铃薯为主的农产品副食化，以杏仁、月饼、毛健茶为主的土特产礼品化。壮大龙头企业。完善“337”工程支持政策，培育10大农业产业化企业（集团）。建设产业基地。打造10个蔬菜标准示范园，改造1000座老旧温室大棚，提高设施农业效益。推动种植业、养殖业、手工业向“一县一业、一村一品”迈进，完善8个省级“一县一业”项目县建设，“一村一品”专业村达到1000个，推动忻定原甜糯玉米、静乐藜麦、神池胡麻等地方特色农业规模化。培育营销主体。大力培育农产品营销公司和经纪人队伍，加快市级农产品电子商务平台建设，推动农产品网上交易，提高农产品销售的信息化水平。创新经营模式。建设农民专业合作示范社省级30家、市级50家、县级80家， 加快发展订单农业，推动诚信经营，不断完善“公司+基地+农户”机制，稳定农民增收预期。

　　加快羊产业的发展。市财政设立5000万元专项资金用于激励养羊业发展，重点支持良繁体系、规模养殖、疫病防控、饲草产业、屠宰加工、市场体系建设和籽粒苋种植推广，力争全年羊饲养量达到700万只以上，在发展大型屠宰和绒毛、羊皮、饲草饲料加工企业上实现新突破，真正把羊产业做成忻州名副其实的大产业。

　　大力推进扶贫开发。全力搞好服务，推动利益共享，加快实施“百企千村”产业扶贫开发工程，以选定的15个项目为示范，形成各类企业投身产业扶贫的生动局面。抓住采煤沉陷区治理新机遇，加快移民搬迁，再搬迁3万人；完善移民小区配套设施，切实提高移民小区的入住率。改进扶贫方式，统筹片区开发、整村推进、教育培训等工作，实施精准扶贫，切实提高扶贫质量，今年再减少贫困人口9万人。

　　扎实办好“五件实事”。改造农村困难家庭危房13000户，改造农村幼儿园30所，推进易地扶贫搬迁，开展乡村清洁工程，启动新型职业农民培训工作。继续为农民办实事、办好事，办一件、成一件，让农民的日子越过越红火。

　　（五）精心实施第三个“大干城建年”，加快推进新型城镇化。

　　完善新型城镇化发展规划。认真落实加快推进新型城镇化若干意见，按照大雁型城镇体系格局，加快“一区四群”城镇体系建设；实施大县城战略，推动县城、集镇、中心村协调发展。坚持“五规合一”，把产业规划放在突出位置，推动产城融合发展。坚决维护规划权威，确保一切建设行为在规划内进行。加强各县（市）规划委员会建设，提高县城规划水平。

　　加快中心城区和县城扩容提质。中心城区完成以改造南北主轴、贯通东部出口，优化城区循环、提升路网功能，强化城市管理、奠定创卫基础为重点的新一轮建设任务。完成“8+22”道路建设与改造工程，做好“五馆一院”和四条特色街建设工作，确保云中河景区“五一”节开放。加快城区西部防洪设施建设。推进国道208线忻州城区改线工程，尽快形成城市路网西环线。抓好城中村改造试点，大力推进城市棚户区集中连片改造。抓好城区道路管网配套和老旧管网改造，新增绿地面积160万平方米。投运忻州汽车客运中心，启动城市公共交通场站建设，完善文化活动场所、便民市场等配套设施，不断提升中心城市的承载服务能力。加快各县（市）新区建设和旧区改造，完成“百镇建设”13个示范镇建设任务。

　　不断提高城市管理水平。深化“三项集中整治”，严厉查处违法建设，依法规范土地建筑市场秩序。抓好各类保障性住房建设，开工1.5万套，基本建成1.47万套。加强建筑质量管理，严格落实建筑质量终身负责制。按照政府许可、市场运作、群众自主、形成合力的原则，创新城市供热、供水、供气、公交等公共产品服务机制，激发各方面参与城市建设的活力。完善数字城管平台，提升城市管理科技水平。深入开展市民教育，大力提升市民素质，积极营造人民城市人民爱的浓厚氛围。

　　完善城镇化健康发展体制机制。鼓励社会资本参与投资运营城市公用设施。深化户籍制度改革，推行居住证制度，落实我省放宽市区、县城和建制镇落户条件要求，促进在城镇稳定就业和生活的常住人口有序实现市民化，不断推进城镇基本公共服务常住人口全覆盖。

　　（六）积极推进生态文明建设，打造宜业宜居的美丽忻州。

　　狠抓节能降耗。继续实行用电量指标与GDP增长挂钩办法，加强重点工业企业节能监察，抓好工业、建筑、交通等领域和煤炭、电力、化工等重点行业节能降耗工作。完善市级能源消费数据库、能源消费量跨区域交易平台，推进企业能效对标。深入开展社会节能，推广应用节能产品和技术设备。强化建筑节能。既有居住建筑节能改造10万平方米、可再生能源建筑应用100万平方米。通过综合施策，确保全市万元GDP能耗指标按季度达进度、按要求完任务。

　　强力推进减排治污。大力推进工业污染源治理，突出抓好燃煤电厂、水泥企业烟气脱硝等环保设施改造，严格控制区域排污总量。加强大气污染防治，实行联防联控，统筹推进扬尘面源控制、燃煤锅炉除尘改造、黄标车淘汰、车用油升级、清洁能源替代等治理。扎实开展忻州城区大气污染集中整治攻坚行动，重点治理城中村、营业性门店等污染源，确保城区大气环境质量明显提升。启动现有污水处理厂提标改造，加快推进乡镇污水处理厂建设。高度重视农村环境问题，突出畜禽养殖污染治理，建成一批农业源减排试点。

　　加快推进造林绿化和生态治理修复工程。深化增绿理水。开展“林业生态建设年”活动，以城区、县城、风景区、旅游点、国省通道为重点，全面推进造林绿化，力争2年内增加林木130万亩。深化集体林权制度改革，落实林权抵押贷款、林地流转等配套措施。抢抓政策机遇，加大京津风沙源治理力度。加强汾河、滹沱河、峨河周边区域排污管理，开展静乐县东碾河等5条中小河流治理，确保地表水和地下水环境安全。完善生态补偿机制，推进采空区、沉陷区、水土流失区生态环境治理修复。

　　切实做好“创模”、“创卫”工作。加快推进城区创建全国环保模范城市、省级园林城市、国家可再生能源应用示范城市“三城同建”工作，启动创卫前期工作。展开5县4镇创卫工作，确保到2015年实现县城和重点镇创建国家卫生城镇全覆盖。推动6县5镇全国卫生城镇完善长效机制，巩固提升创卫成果。扎实推进各县（市）创建省级环保模范城工作。

　　加强环境监测和环保执法。加快市级监控平台升级改造，健全监测网络，搞好PM2.5监测发布，积极回应社会关切。保持高压治污态势，加大环保执法力度，有案必查、违法必处，确保不发生重大环境事故和环境事件。

　　（七）高度重视民生保障，统筹推进教育文化社会事业协调发展。

　　千方百计扩大就业。实施更加积极的就业政策，统筹做好高校毕业生、农村转移劳动力、城镇困难人员、退役军人等各类人群的就业工作。加大就业岗位开发力度，加强政府公益性岗位就业管理。落实就业援助措施，提高就业技能培训针对性和实效性，积极帮助困难人员就业。开展创业型城市创建活动，以创业带动就业。

　　促进教育事业优先发展。统筹城乡义务教育资源均衡配置，加快农村义务教育薄弱学校改造，实施义务教育学校标准化建设，实现义务教育均衡发展目标。大力发展学前教育，新建、改扩建15所标准化公办幼儿园，改造30所农村幼儿园。启动忻州一中北校区建设等项目前期工作，扩大优质教育资源覆盖面。加快发展现代职业教育，抓好实训基地建设。实施特殊教育提升计划，推进继续教育改革发展。加强教师队伍建设和师德师风建设，充实农村教师队伍，落实边远贫困地区教师待遇。健全家庭困难学生资助体系，努力促进教育公平。不断规范办学行为，进一步解决中小学择校、城镇学校大班额问题。切实减轻学生课业负担，促进学生身心健康。

　　着力增加居民收入。认真执行最低工资制度，加强企业职工工资调控，推动规模以上企业工资集体协商全覆盖。加强监督检查，确保机关事业单位人员工资和津贴补贴正常发放。支持农民以承包经营权入股实现农业产业化，鼓励农村发展合作经济，健全农民工工资支付保障机制，多渠道增加农民收入。着力稳定市场物价，努力保障群众生活。

　　切实搞好社会保障。继续扩大城镇职工和城乡居民养老、医疗、失业、工伤、生育等各项保险覆盖面。整合城乡居民基本养老保险制度和城乡居民医疗保险制度。严格城乡居民最低生活保障管理。企业退休人员基本养老金提高10%，城镇居民基本医保和新农合人均财政补助标准提高40元，城乡低保标准每人每月分别提高25元、22元。提高农村五保对象供养补助标准，建立一级重度残疾人护理补贴和贫困一级重度残疾人生活补贴制度。扩大种植业、养殖业等农业保险覆盖面，有效防范农业风险。

　　加快文化改革发展。深化文化体制机制改革，促进转企改制国有文艺院团改革发展，推动经营性文化单位完善法人治理结构。认真践行社会主义核心价值观，深化文明创建活动。坚持正确舆论导向，加强网络文化建设和管理，不断提高应对网络舆情的能力。完善城乡公共文化服务体系，继续组织好“周末大戏台”、“乡村大舞台”等文化活动，实施好“送戏下乡”和“文化惠民”工程。加快发展文化创意、广告会展、数字动漫等新兴产业，扶持发展移动多媒体、网络视听、数字出版等潜力产业，打造一批骨干文化企业，加快向文化产业大市迈进。加强文物和非物质文化遗产保护，依法打击破坏和盗窃文物等违法犯罪行为。发展体育事业，支持群众性体育活动，推动全民健身运动广泛发展，努力提升竞技体育水平。

　　大力发展卫生事业。健全全民医保体系，加强疾病预防控制，完善重特大疾病医疗保险和救助制度。完善社区卫生服务体系，推进城乡公共卫生服务均等化。巩固和发展县级公立医院综合改革成果，确保市人民医院投入运行，开工建设市儿童医院和早教中心。加强全科医生培养，鼓励社会办医。全面做好人口计生工作，实施一方是独生子女的夫妇可生育两孩政策。

　　加强和创新社会治理。完善信访工作制度，有效预防和化解社会矛盾。推进城镇社区网格化管理，探索农村社区管理新模式，发挥人民调解员作用，夯实社会治理基层基础。加大食品药品安全监管力度，加快建设食品全过程、全节点、全方位监控体系，确保“舌尖上的安全”。加强应急管理和防灾减灾能力建设，妥善应对各类突发事件。高度重视禁毒工作，推进社区戒毒机制建设。加强社会治安综合治理，严防严惩各类违法犯罪活动，扎实推进平安忻州建设。

　　（八）坚持不懈抓好安全生产，为各项工作奠定基础。

　　完善四个体系。完善政府监管责任体系。坚持把政府监管责任放在第一位，切实做到综合监管、部门监管、专门监管、行业监管、属地监管的全覆盖、全天候、立体化。完善企业主体责任体系。确保各企业做到有安全生产机构，有专职安全人员，有安全管理制度，有安全防控措施，经常性组织安全培训，对安全生产工作实行严格考核。完善岗位责任体系。全面落实“一岗双责”要求，真正形成层层关注、人人过问、互相监督、齐抓共管的有效机制。完善应急体系。做到应急预案、应急设施、应急演练“三落实”。

　　严格四项管理。严格安全风险预控管理。全面排查和识别各类危险源，落实管控责任，确保对本地本部门本单位的重大危险源做到情况清、底数明。严格全员安全管理。深入开展员工“知责、履责、尽责”活动，把安全生产责任落实到每位员工身上、每个具体环节。严格值班管理。落实值班要求，及时报告、解决有关安全生产问题。严格安全档案管理。健全安全生产记实档案，让一切言行都有迹可循，实行终身负责制。

　　坚持三项制度。坚持警示教育制度。推进安全警示教育进学校、进家庭、进社区、进机关，开展“人人都是安全员”活动，真正使安全生产知识入心入脑。坚持监督举报制度。调动全社会参与安全监督的积极性，重视发挥和利用好网络监督的作用，鼓励群众对安全隐患、对安全生产不作为现象进行举报，最大限度压缩事故隐患的空间。坚持重奖重罚制度。重奖安全生产工作先进单位，重罚安全生产工作落后单位，通过重奖重罚，建立良好导向。

　　强化专项整治。深化安全生产大检查，切实做到全覆盖、零容忍、严执法、重实效。突出抓好煤矿、非煤矿山、尾矿库、道路交通、危险化学品、民爆物品、特种设备、建筑施工、消防、水库、学校、商场等重点行业、重点领域安全生产，夯实安全生产基础。强化从严问责，坚持关口前移，坚持全程公开，以铁的纪律抓好安全生产，坚决杜绝重特大事故，有效遏制较大事故，努力减少一般事故，推动全市安全生产形势向稳定好转坚实迈进。

　　（九）全面深化改革，切实激发转型跨越的动力和活力。

　　深化行政审批制度改革。进一步简政放权，认真做好审批权限下放的承接工作，继续清理市级行政审批项目。进一步优化审批流程，公布市级政府行政审批目录，提高行政效率。进一步加强监督，坚持“接”、“放”、“管”结合，搞好指导监督。

　　完成政府机构改革。完善政府机构职能配置，完成市县两级政府机构改革任务。严格控制机构编制，财政供养人员只减不增。

　　深化企业改革。继续坚定不移、积极稳妥地推进市属国有企业的改革。完善忻通公司、水务集团的法人治理机构，完成钨丝厂、云河集团、云马焦化厂改制的后续工作，启动煤机厂、酒厂和玻璃瓶厂等企业改制工作。鼓励各县（市、区）大力推进国有企业改革。推动民营企业建立现代企业制度，支持民营企业做大做强。

　　扎实推进事业单位分类改革。严格分类管理，科学界定负责行政职能、公益服务、生产经营活动事业单位的类别。严格编制管理，确保全市事业编制总额控制在2012年底总量内。积极稳妥地推进自收自支事业单位改革，坚决防止以改革为名把不符合规定的事业单位纳入财政供养范围。

　　深化农村改革。切实做好农村土地承包经营权确权登记颁证试点工作，加快建立和完善县乡村三级土地流转服务机制、纠纷调处机制、风险保障机制，有序推进农村土地流转，发展多种形式规模经营。鼓励发展专业大户、家庭农场、农民合作社、农业企业等新型农业经营主体。

　　深化工商登记制度改革。推进公司注册资本认缴制，实行企业年检改年报和先照后证，为各类企业发展创造良好环境。

　　（十）进一步加强政府自身建设，建设人民满意政府。

　　坚持执政为民。深入开展政府系统群众路线教育实践活动。认真落实全市政府系统工作人员“五章约法”。牢固树立过紧日子的思想，坚持开源节流、量入为出、勤俭节约、严格管理，做好“三公”经费预决算公开工作，不断提高财政资金的使用效益。

　　严格依法行政。认真执行市人大及其常委会的决议，支持市政协履行职能，认真听取各民主党派、工商联、无党派人士的意见，充分发挥工会、共青团、妇联等人民团体作用。完善科学民主决策机制，增强政府工作的透明度和公众参与度。提高运用法治思维和法治手段解决问题、推动工作的能力，使政府工作严格在法律和政策的范围内进行。

　　加快职能转变。处理好政府与市场的关系，充分发挥市场在资源配置中的决定性作用，更好发挥政府作用，切实管好政府该管的事，努力建设服务型政府。坚持依法行政，协同法检两院，形成依靠法治推动工作的合力。处理好与基层的关系，尽力调动县、乡、村的积极性。

　　提高工作水平。大兴学习之风，推进学习型机关建设，切实提升政府工作人员的法律政策水平和履职尽责能力。改进调查研究，确保政府决策符合实情、满足需要。充分发挥市场、科技、网络的作用，创新行政管理方式。

　　加强廉政建设。认真执行《党政机关厉行节约反对浪费条例》和国务院“约法三章”。深入推进反腐倡廉，加快建立廉政风险防控体系。严格收支两条线管理，做到应收尽收，杜绝跑冒滴漏。严格执行《招投标法》和集中采购制度，加大向社会购买事务性服务力度。深化政务公开，更好发挥审计监督作用。加强行政监察，纠正行业不正之风，严肃查处政府工作人员违法违纪行为，确保干部清正、政府清廉。

　　带头改进作风。认真执行中央八项规定和省市实施办法，改进文风会风，坚决反对“四风”，做到令行禁止，确保政令畅通。自觉接受社会、群众、媒体监督，坚决查处不作为、慢作为、乱作为的人和事，坚决整治政府机关“庸、懒、散”现象，努力建设人民信赖的法治政府、为民政府、服务政府、效能政府、诚信政府、廉洁政府！

　　各位代表！人民赋予重托，奋斗创造未来。让我们在市委的正确领导下，高举中国特色社会主义伟大旗帜，认真实施“3581”发展战略，咬定目标，创新攻坚，真抓实干，锐意进取，为建设富裕文明、开放和谐、充满活力的新型工业旅游城市而努力奋斗！
